# Predicting COVID‐19 booster vaccine intentions

**DOI:** 10.1111/aphw.12349

**Published:** 2022-02-22

**Authors:** Martin S. Hagger, Kyra Hamilton

**Affiliations:** ^1^ Department of Psychological Sciences University of California Merced California USA; ^2^ Health Sciences Research Institute University of California Merced California USA; ^3^ Faculty of Sport and Health Sciences University of Jyväskylä Jyväskylä Finland; ^4^ School of Applied Psychology Griffith University Brisbane Queensland Australia; ^5^ Menzies Health Institute Queensland Griffith University Brisbane Queensland Australia

**Keywords:** behavior change, integrated models, social cognition theory, vaccine attitudes and beliefs, vaccine hesitancy

## Abstract

Achieving broad immunity through vaccination is a cornerstone strategy for long‐term management of COVID‐19 infections, particularly the prevention of serious cases and hospitalizations. Evidence that vaccine‐induced immunity wanes over time points to the need for COVID‐19 booster vaccines, and maximum compliance is required to maintain population‐level immunity. Little is known of the correlates of intentions to receive booster vaccines among previously vaccinated individuals. The present study applied an integrated model to examine effects of beliefs from multiple social cognition theories alongside sets of generalized, stable beliefs on individuals' booster vaccine intentions. US residents (*N* = 479) recruited from an online survey panel completed measures of social cognition constructs (attitude, subjective norms, perceived behavioral control, and risk perceptions), generalized beliefs (vaccine hesitancy, political orientation, and free will beliefs), and COVID‐19 vaccine intentions. Social cognition constructs were related to booster vaccine intentions, with attitude and subjective norms exhibiting the largest effects. Effects of vaccine hesitancy, political orientation, and free will beliefs on intentions were mediated by the social cognition constructs, and only vaccine hesitancy had a small residual effect on intentions. Findings provide preliminary evidence that contributes to an evidence base of potential targets for intervention messages aimed at promoting booster vaccine intentions.

## INTRODUCTION

The COVID‐19 pandemic has contributed substantially to excess deaths worldwide in 2020 and 2021 (Centers for Disease Control and Prevention [CDC], 2021b). While COVID‐19 infections result only in mild respiratory symptoms in the vast majority of cases, a substantive minority develop into serious, potentially life threatening cases requiring hospitalization, particularly among older adults, individuals with comorbid conditions, and the immunocompromised (Clark et al., 2020). This has placed a substantive burden on healthcare provision and cost (Bartsch et al., 2020). With COVID‐19 cases continuing to rise in many regions of the world largely due to the emergence highly contagious variants, developing wide‐scale immunity among the population through the vaccination is likely to be a cornerstone strategy in reversing infection rates and bringing the pandemic under control (Kashte et al., 2021). Rollout of COVID‐19 vaccines has largely been successful with substantive proportions of populations in high‐income countries reaching vaccination rates of 60 per cent or greater, although rates lag in many countries (Mathieu et al., 2021). However, given emerging evidence that immunity afforded by the current vaccines may wane over time (Naaber et al., 2021), governments in a number of countries have authorized administration of “booster” doses of the vaccine to maintain immunity levels (e.g. Bar‐On et al., 2021; CDC, 2021a). These authorizations have been made in advance of comprehensive evidence for the efficacy and safety of booster vaccines but have been authorized on an emergency basis given the severity of rates of infection and continued waves of infection and based on preliminary evidence of their efficacy and expert opinion (Bar‐On et al., 2021; Mattiuzzi & Lippi, 2022).

In addition, inception and rollout of booster vaccine programs has occurred in the backdrop of slowing rates for initial COVID‐19 vaccination in the general population, with rates of increase in full vaccination shallowing and, in some regions, plateauing (Lukpat, 2021). These trends have led to concerns that vaccination rates may fall short of affording the widespread population‐level immunity required to keep COVID‐19 infections under control. These trends highlight not only the imperative of ongoing promotion of first‐time vaccination but also maximization of uptake of booster vaccines to maintain immunity among the previously vaccinated, particularly among vulnerable groups (Sarpatwari et al., 2021). To do so, governments and healthcare providers need to ensure effective interventions that promote booster vaccine uptake. Such interventions necessitate a fundamental understanding of the correlates of individuals' intention to get vaccinated with a booster vaccine. As a consequence, researchers need to consider the application of theories from behavioral science to identify constructs that reliably inform individuals' decisions to get the booster vaccine, consistent with previous research on initial vaccine uptake (e.g. Caserotti et al., 2021; Guillon & Kergall, 2021; Tong et al., 2021). This endeavor has value as it may flag potentially modifiable constructs that could be targeted by behavior change strategies such as persuasive messaging in interventions aimed at promoting booster vaccine uptake (e.g. Hamilton & Johnson, 2020). Importantly, basing behavioral interventions on theory has been associated with their optimal efficacy and efficiency in changing behavior (e.g. McEwan et al., 2019).

Theories of social cognition are a prominent class of theory that has typically been adopted to predict health behavior (Conner & Norman, 2015) including vaccine‐related behaviors, such as intentions to get the influenza vaccine (e.g. Ng et al., 2020), and may be highly pertinent in the context of predicting booster vaccine intentions. Theories such as the theory of planned behavior (Ajzen, 1991) and the health action process approach (Schwarzer et al., 2008) have identified attitudes, subjective norms, perceived behavioral control or self‐efficacy, and risk perceptions as key constructs that are related to health behavior participation. These constructs reflect individuals' subjective judgments on the utility, social pressure from significant others, personal ability and capacity, and personal risk, respectively, with respect to performing a given target health behavior at some point in the future. Meta‐analytic research has supported effects of these theoretical constructs in predicting intentions to engage in health behaviors across multiple populations, contexts, and behaviors (e.g. McEachan et al., 2011; Zhang et al., 2019). They have also been applied to COVID‐19 mitigation behaviors (e.g. Hamilton et al., 2020) and, critically, to intentions to get a COVID‐19 vaccine (e.g. Caserotti et al., 2021; Guillon & Kergall, 2021). Consistent with theory and the prior research, these social cognition beliefs would be expected to be key correlates of intentions to receive a subsequent COVID‐19 booster vaccine among previously vaccinated individuals.

Alongside belief‐based determinants from social cognition theories, researchers have also identified other individual difference constructs and generalized beliefs with high stability that may predict health behavior intentions (e.g. Bogg & Milad, 2020; Hagger et al., 2019; Kekäläinen et al., 2022). These constructs encompass personality traits, as well as sets of more stable beliefs such as beliefs in self‐control and free will. Researchers have augmented existing social cognition theories to account for the effects of these constructs on intentions and behavior in health contexts and have conceptualized them as distal determinants that relate to intentions and behavior mediated by the belief‐based constructs from the theories such as attitudes and self‐efficacy (e.g. Bogg & Milad, 2020; Hagger et al., 2019). In terms of mechanism, such constructs are conceptualized as sources of information from which individuals draw when estimating their beliefs with respect to performing the target health behavior in future (Ajzen, 1991; Kaushal et al., 2021; Protogerou et al., 2018). Research has corroborated the predictions of these integrated social cognition theories in health behavior contexts. For example, studies have demonstrated indirect effects of individual difference constructs such as personality and generalized beliefs on intentions toward, and actual participation in, health behavior, mediated by social cognition constructs (e.g. Bogg & Milad, 2020; Hagger et al., 2019).

In the context of COVID‐19 vaccination behavior, a slate of individual difference and generalized beliefs have been identified as potentially salient correlates of intentions to get vaccinated (e.g. Debus & Tosun, 2021; Sherman et al., 2021). Most prominent of these is vaccine hesitancy, defined as individuals' stated expectation to delay or refuse to receive a vaccine (MacDonald, 2015). Vaccine hesitancy is endemic in a substantive minority of individuals in the context of COVID‐19 vaccines, and it is suggested to be a key contributor to the slowing of COVID‐19 vaccination rates (e.g. Sallam, 2021). Vaccine hesitancy likely reflects numerous beliefs and concerns that individuals hold that affect their motivation or intention to get vaccinated including fears over insufficient testing and rapid development of the vaccines; a lack of trust in government public health systems and vaccine producers and political beliefs reflecting antigovernment sentiment; beliefs that the vaccine represents legislative overreach and an undermining of personal rights; general fears over the vaccine process such as a fear of needles; complacency and lack of perceived risk; and low confidence in gaining access to the vaccine (e.g. Freeman et al., 2020, 2021; Jennings et al., 2021). Studies have indicated that vaccine hesitancy is a salient determinant of individuals' intentions to get vaccinated (e.g. Guillon & Kergall, 2021). Vaccine hesitancy is also likely to be an important ongoing determinant of intentions to get a booster vaccine among those who have previously been vaccinated, because many of those who chose to get an initial COVID‐19 vaccine still held skeptical views with respect to the vaccine and its efficacy but ultimately decided to get vaccinated (Siegler et al., 2021). Assuming these links, we therefore hypothesized that relations between vaccine hesitancy and booster vaccine intentions will be mediated by individuals' attitudes and beliefs with respect to getting the booster vaccine.

In addition, individuals' attitudes and beliefs toward vaccines in the context of the COVID‐19 pandemic are likely to be affected by their political and free will beliefs. The COVID‐19 pandemic has occurred in political climates in which populist right‐wing movements and “anti‐vax” groups and influencers are highly visible with ready access to social media forums to promulgate their views (e.g. Stecula & Pickup, 2021). These perspectives have contributed to public mistrust of authorities in a substantive minority, manifested in concerns over the rapid development of vaccines and general dissatisfaction with government mandates to curb COVID‐19 infections (Jennings et al., 2021). Research has indicated that individuals with more conservative, right‐leaning political views are more likely to cite skepticism over COVID‐19 vaccines and less likely to state positive vaccination intentions and get vaccinated (e.g. Bilewicz & Soral, 2021; Brinson, 2022). Consistent with this prior research, political ideology would be expected to impact individuals' intentions to receive a booster vaccine, such that individuals with more conservative orientation would be less likely to intend to get a booster vaccine.

There has been considerable recent interest in the role of individuals' free will beliefs as correlates of health‐related behavior. Free will beliefs reflect individuals' generalized endorsement of the extent to which they are able to affect outcomes in their social world. Free will beliefs therefore reflect individuals' beliefs in their capacity for agency, self‐regulation, personal choice, and control over their actions and decision‐making (Baumeister & Monroe, 2014). Free will beliefs are conceptualized as a generalized, enduring construct, similar to personality and other dispositional constructs, and, therefore, tend not to refer to specific behavior contexts. As a dispositional construct, free will beliefs are expected to relate to multiple actions and decisions in behavioral contexts where volition and decision‐making are front and center. For example, individuals endorsing free will beliefs have been shown to be more likely to engage in actions that are adaptive and conducive to better functioning (e.g. more cohesive social interactions and better performance in educational and occupational contexts) (Baumeister & Monroe, 2014). In the domain of health, individuals endorsing free will beliefs have been shown to be more inclined to report greater well‐being and be proactive in taking action to achieve their goals and engage in adaptive behaviors (Crescioni et al., 2016).

Extending these findings, free will beliefs are expected to be highly relevant to vaccination decisions in the context of COVID‐19. Given findings that individuals with stronger free will beliefs are more proactive in engaging in health‐promoting behaviors, we expect individuals endorsing these beliefs would be more likely to form an intention to get a booster vaccination. Furthermore, it is unlikely that individuals endorsing a belief in free will would intend not to accept a booster vaccine given the consequences are likely to present a considerable health risk (Baumeister & Monroe, 2014). However, this prediction may be at loggerheads with evidence suggesting that those expressing vaccine hesitancy view vaccines, and the programs that administer them, as restrictions on personal freedoms, which may be inconsistent with their free will beliefs (Piltch‐Loeb et al., 2021). Nevertheless, given evidence that individuals that tend to endorse free will beliefs tend to prioritize their health, and, as a consequence, are likely to proactively seek out knowledge on the benefits of health behavior such as vaccination, we expect them to be more likely to form positive beliefs with respect to future vaccination behavior and form intentions to do so.

Taken together, we predict that political and free will beliefs are likely to be important generalized correlates of individuals' beliefs and intentions toward getting a booster vaccine. We predict that attitudes, subjective norms, and perceived behavioral control will mediate effects of political orientation and free will beliefs on intentions. This is because individuals' dispositional beliefs provide important intraindividual information on which individuals rely when estimating their beliefs about specific future behaviors (Ajzen, 1991). Individuals prompted to estimate their beliefs with respect to their future booster vaccination behavior will likely draw, consciously or nonconsciously, from their political and free will beliefs, when doing so. For example, individuals harboring generalized antigovernment sentiment due to their political beliefs and relate that to the vaccine development and efficacy are likely to express negative attitudes toward the booster vaccine. Analogously, individuals with strong free will beliefs are likely to seek out actions that promote health and strategically align their dispositions with beliefs relevant to performing health‐promoting behaviors like vaccination (e.g. positive attitudes and perceptions of control). Consistent with the predictions of the theory of planned behavior, these beliefs are those that contribute to intention formation, so we expect indirect effects of these dispositional variables on booster vaccination intentions mediated by the sets of beliefs that underpin the intentions, namely, attitudes, subjective norms, and perceived behavioral control.

## THE PRESENT STUDY

The purpose of the present study was to identify the correlates of intentions to get a COVID‐19 booster vaccine when offered among individuals who had previously been fully vaccinated. Specifically, we applied an integrated model to examine the extent to which social cognition constructs derived from multiple theories applied in health behavior contexts explained variance in stated intentions to receive the booster vaccine alongside additional individual difference and dispositional variables, namely, vaccine hesitancy, political orientation, and free will beliefs. Consistent with social cognition theories (e.g. Ajzen, 1991; Fishbein et al., 2001), our model specified that individuals' booster vaccine intentions would be a function of their attitudes, subjective norms, perceived behavioral control, and risk perceptions with respect to getting the booster vaccine. Further, consistent with integrated theories examining individual differences and dispositional constructs in social cognition theories (Bogg & Milad, 2020; Hagger et al., 2019; Hagger & Hamilton, 2020; Ng et al., 2020), we predicted that the social cognition constructs would mediate the effects of vaccine hesitancy, political orientation, and free will beliefs, on booster vaccine intentions. We therefore expected nontrivial indirect effects of the individual difference and dispositional constructs on booster vaccine intentions through the social cognition constructs. Confirmation of these indirect effects would provide preliminary evidence that the variance individual difference and dispositional constructs share with booster vaccine intentions is accounted for by the belief‐based constructs directly implicated in decision‐making according to social cognition theories. Such data may indicate possible reasons why enduring beliefs are related to vaccine intentions. We expected our model test to contribute the first data to an evidence base of potential targets for behavioral interventions aimed at promoting booster vaccine intentions in the context of COVID‐19.

## METHOD

### Participants and design

The present study adopted a cross‐sectional correlational survey design. Participants were COVID‐19 vaccinated US residents (*N* = 479, 56.8% female) recruited from an online research panel. To be eligible for inclusion, participants had to be aged 18 years or older and have reported receiving both doses of a two‐dose (i.e. Moderna, Pfizer/BioNTech), or one dose of a single‐dose (i.e. Johnson & Johnson/Janssen), FDA/CDC‐approved COVID‐19 vaccine. Participants were also screened for age, sex, and geographical region, and quotas were imposed during recruitment to ensure that the final samples closely matched the national distributions for these characteristics in the US as a whole. However, while the recruited sample closely matched the US population on the aforementioned demographic variables, it should not be considered representative given that quotas were not placed on other characteristics such as race and socio‐economic status. Data on response rate were not collected. Data were collected in May 2021.

### Procedure

Eligible US residents approached by the panel company to participate in the study were informed that they were being asked to participate in a survey on COVID‐19 vaccines. They were subsequently provided with information outlining study requirements. They were also informed of their right to decline participation at any point and to have their data deleted. They were then required to provide opt‐in consent to participate prior to advancing to the survey. Consenting participants completed self‐report measures of social cognition constructs from the proposed integrated social cognition model (attitude, subjective norm, perceived behavioral control, and risk perceptions) and intentions with respect to receiving a booster shot for the COVID‐19 vaccine when offered and measures of COVID‐19 vaccine hesitancy, political orientation, and free will beliefs. Participants also self‐reported a series of sociodemographic variables. Data were collected using the Qualtrics™ online survey tool. Approval for study procedures was granted prior to data collection from the Griffith University Research Ethics Committee (Reference #2021/108).

### Survey measures

#### Social cognition constructs

Multi‐item measures of the attitude, subjective norm, perceived behavioral control, risk perceptions, and intention constructs from the proposed integrated model were developed according to published guidelines (Ajzen, 1991; Schwarzer et al., 2008) with responses provided on 7‐point response scales. Each measure referenced the target behavior of receiving the COVID‐19 booster vaccine when offered according to the national COVID‐19 vaccination program. Complete study measures are provided in Appendix A in the Supporting Information.

#### Vaccine hesitancy

Vaccine hesitancy was measured using a single item (“Overall, how hesitant are you about getting the COVID‐19 vaccine?”) adapted from previous measures (Freeman et al., 2021), with responses provided on a 7‐point scale (1 = *not at all* to 7 = *very much*). Higher scores indicated a higher level of vaccine hesitancy.

#### Political orientation

Participants reported their political orientation on two items in which they were prompted to place themselves on a political beliefs continuum (Kroh, 2007). Responses were made on slider scale (0 = *far left/strongly progressive* to 100 = *far right/strongly conservative*) with higher scores representing greater endorsement of conservative, right‐leaning political values.

#### Free will beliefs

Free will beliefs were measured using the five‐item “free will” subscale of the Free Will Inventory (e.g. “People always have free will”; Nadelhoffer et al., 2014). Responses were provided on 5‐point scales (1 = *strongly disagree* to 5 = *strongly agree*) with higher responses reflecting greater free will beliefs.

#### Sociodemographic variables

Participants self‐reported the following sociodemographic variables: age in years, sex (male, female, and nonbinary), employment status (currently unemployed/full‐time caregiver, part‐time/casual employed, currently employed full‐time, leave without pay/furloughed, and retired), race/ethnicity (Black, Caucasian/White, Asian, Middle Eastern, and other), annual household income, highest level of formal education (completed junior/lower/primary school, completed senior/high/secondary school, postschool vocational qualification/diploma, undergraduate university degree, and postgraduate university degree), previous diagnosis for COVID‐19 (yes/no), vaccine type (Johnson & Johnson/Janssen, Moderna, and Pfizer/BioNTech) and number of shots, and received an influenza vaccine in the previous year (yes/no). Categorical income (low, middle, and income), highest education level (completed school education only vs. completed postschool education), and race (Caucasian/White vs. non‐White) variables were computed for use in subsequent data analyses.

### Data analysis

Prior to formal analysis, we computed manifest scales for each social cognition and dispositional construct by averaging the item scores for each. We also generated descriptive statistics for the sociodemographic variables, McDonald's (1999) omega (*ω*) reliability statistics for the social cognition and dispositional construct scales, and zero‐order correlations coefficients among all study variables. Hypothesised relations among the integrated model constructs according to our integrated model were tested in a single‐indicator structural equation model using the lavaan analysis package implemented in R (Rosseel, 2012). Single‐indicator structural equation models use scale reliabilities to provide an estimate of the measurement error of each variable in the model. Single‐indicator models are a good choice for complex models and smaller sample sizes because they minimize parameterization but yield parameter estimates that compare extremely favorably with models using full indicator latent variables (Savalei, 2019). In our model, relations between social cognition constructs (attitude, subjective norm, perceived behavioral control, and risk perceptions) and COVID‐19 booster vaccine intentions, and between the individual difference and dispositional variables (vaccine hesitancy, political orientation, and free will beliefs) and each social cognition construct, were set as free parameters. In addition, free parameters for relations between the individual difference and dispositional variables and intentions were also estimated. In addition, age, sex, education level, employment status, ethnicity, previous COVID‐19 diagnosis, and previous influenza vaccine were included as covariates in the model by setting relations between these variables and intentions as free parameters. Income was not included as a covariate due to a high degree of missing data as participants could opt out of reporting their income for ethical reasons.

Given the goodness‐of‐fit chi‐square (*χ* ^2^) comparing researcher‐imposed models with the fully saturated model in structural equation modeling is typically oversensitive to misfit, we used recommended incremental fit indices to estimate our model fit including the comparative fit index (CFI), the Tucker–Lewis index (TLI), the standardised root mean‐squared of the residuals (SRMSR), and the root mean square error of approximation (RMSEA) and its 90 per cent confidence interval (90% CI). Values for the CFI and TLI should approach or exceed .95, values for the SRMSR should be less than or equal to .08, and values for the RMSEA should be below .06 with a narrow 90 per cent CI (Hu & Bentler, 1999).

## RESULTS

### Participants

Sample characteristics are presented in Table 1. Almost half of participants were female (56.78%) and reported being currently employed (47.81%). The majority of participants were White/Caucasian (87.89%), from high (52.40%) and middle (14.41%) income backgrounds, and had completed a postschool qualification or higher education degree (73.69%). Relatively few participants had received a COVID‐19 diagnosis previously (7.93%), all were fully vaccinated with nearly half receiving the Pfizer/BioNTech two‐dose vaccine (48.64%), and approximately one third reported getting an influenza vaccine in the previous year (33.61%).

**TABLE 1:** Sample characteristics and descriptive statistics

Variable	Statistics		Variable	Statistics	
Participants, *N*	479		Education level, *n* (%)		
Age, *M* years (*SD*)	52.14	(14.55)	Completed junior/lower/primary school	4	(0.83)
Gender, *n* (%)			Completed senior/high/secondary school	122	(25.47)
Female	272	(56.78)	Postschool vocational qualification/diploma	66	(13.78)
Male	203	(42.38)	Undergraduate University degree	171	(35.70)
Nonbinary	2	(0.42)	Postgraduate University degree	116	(24.22)
Not specified/prefer not to answer	2	(0.42)	Previous diagnosis for COVID‐19		
Employment status, *n* (%)			Yes	38	(7.93)
Currently unemployed/full‐time caregiver	78	(16.28)	No	440	(91.86)
Part‐time/casual employed	44	(9.19)	Prefer not to say	1	(0.21)
Currently employed full‐time	229	(47.81)	Current COVID‐19		
Leave without pay/furloughed	1	(0.21)	Yes	18	(3.76)
Retired	127	(26.51)	No	460	(96.03)
Race, *n* (%)			Prefer not to answer	1	(0.21)
Black	19	(3.97)	Vaccine type		
Caucasian/White	421	(87.89)	Pfizer/BioNTech	233	(48.64)
Asian (South‐East Asia/South Asia)	22	(4.49)	Moderna	174	(36.33)
Middle Eastern	1	(0.21)	Johnson & Johnson	62	(12.95)
Other	13	(2.71)	Not known	5	(1.04)
Prefer not to answer	3	(0.63)	Prefer not to say	5	(1.04)
Income, *n* (%)^a^			Number of vaccine shots to date		
Low income (≤US$30,000)	73	(15.24)	Two	427	(89.14)
Middle income (US$30,001 to $50,000)	69	(14.41)	One	51	(10.65)
High income (>US$50,000)	251	(52.40)	Not specified	1	(0.21)
Prefer not to answer	86	(17.95)	Influenza vaccine in the previous year		
			Yes	161	(33.61)
			No	318	(66.39)

^a^ Participants were given the choice of opting out of reporting their income.

### Preliminary analyses

Scales for the social cognition constructs, intention, political orientation, and free will beliefs exhibited adequate reliability coefficients (*ω* range = .868 to .976). Overall, participants reported positive beliefs (attitude, subjective norms, and perceived behavioral control) and intentions toward getting the booster vaccine, and high free will beliefs, indicated by mean scale scores for these constructs above the scale midpoint. Participants also reported relatively low levels of risk perceptions and vaccine hesitancy; mean scale scores for these constructs fell below the midpoint.

Correlations indicated statistically significant, positive correlations among the attitude, subjective norms, perceived behavioral control, intention, and free will beliefs constructs (*r* range = .180 to .755, *p*s < .001). Attitude, subjective norms, perceived behavioral control, and intention were significantly and negatively correlated with risk perceptions, vaccine hesitancy, and political orientation (*r* range = −.159 to −.492, *p*s < .01). Vaccine hesitancy was significantly and positively correlated with political orientation (*r* = .236, *p* < .001) and risk perceptions (*r* = .521, *p* < .001). Free will beliefs were significantly and positively correlated with political orientation (*r* = .242, *p* < .001) but not with risk perceptions and vaccine hesitancy. Correlations of social cognition, intention, and dispositional constructs with sociodemographic variables revealed few statistically significant associations. The most consistent relations were between the attitude, subjective norms, perceived behavioral control, and intention constructs, and receipt of a prior influenza vaccine (*r* range = .137 to .185, *p*s < .003). In addition, intention was also significantly and positively correlated with highest education level (*r* = .114, *p* = .013), indicating that individuals who attained a higher level of education were more likely to report an intention to get a booster vaccine. By contrast, none of the other sociodemographic variables were significantly correlated with intentions.

We also found no systematic differences in the social cognition and dispositional variables by income level (low, medium, and high). Although a MANOVA revealed an overall significant difference (Wilks' *Λ* = 0.929, *F*(16,390) = 1.800, *p* = .029, *η* ^2^ = 0.036), follow‐up tests revealed a significant difference on the subjective norm construct only with a small effect size (*F*(2,390) = 5.206, *p* = .006, Cohen's *d* = 0.211). This is an important finding given we did not include income as a covariate in our analysis. Full descriptive statistics and correlations are reported in Appendices B and C in the Supporting Information, respectively.

Vaccine dosing regimen may affect individuals' perceptions of risk. For example, individuals receiving a vaccine with a one‐dose regimen (i.e. the Johnson & Johnson/Janssen vaccine) may believe they have better protection against COVID‐19 infection and report lower risk perceptions as a result than those on a two‐dose regimen (i.e. Moderna and Pfizer/BioNTech vaccines). To examine this premise, we tested whether vaccine type was related to individuals' beliefs. We conducted a MANOVA with the social cognition and dispositional variables, including risk perceptions, as multiple dependent variables and vaccine type (“one shot” vs. “two shot” regimens) as the independent variable. The MANOVA flagged an overall significant difference (Wilks' *Λ* = 0.963, *F*(8,467) = 2.200, *p* = .025, *η* ^2^ = 0.037). Univariate follow‐up tests revealed a significant difference in risk perceptions across vaccine regimen (*F*(1,467) = 9.976, *p* = .002, Cohen's *d* = 0.292). Participants on a one‐shot regimen were more likely to have lower risk perceptions than those on a two shot regimen. There were no significant differences in booster vaccine intentions across regimen type (*F*(1,467) = 3.874, *p* = .050, *d* = 0.182). These findings indicate differences in risk perceptions in those receiving different vaccine regimens, but this was not reflected in intentions to get a booster vaccine.

### Structural equation model

**FIGURE 1:**
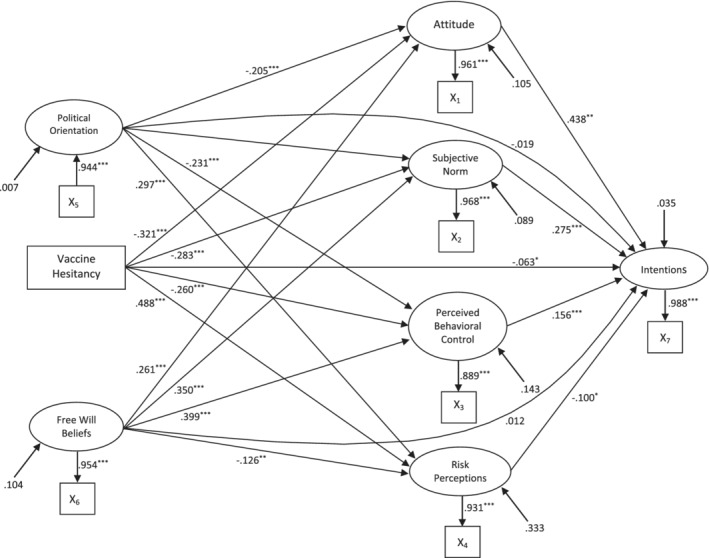
Standardised parameter estimates from the single‐Indicator structural equation model of the proposed integrated model predicting COVID‐19 booster vaccine intentions. ^***^ *p* < .001; ^**^ *p* < .01; ^*^ *p* < .05

The proposed model exhibited adequate fit with the data according to multiple goodness‐of‐fit indices (CFI = .977, TLI = .913, SRMSR = .035; RMSEA = .054, 90% CI [.038, .071]). Standardised parameter estimates from the proposed model are summarized in Figure 1, and parameter estimates and confidence intervals for the factor loadings, and the direct and indirect effects, in the model are summarized in Table 2.^1^ Consistent with predictions, attitude, subjective norm, and perceived behavioral control were statistically significant, positive predictors of booster vaccine intentions, with small‐to‐medium effect sizes. Risk perceptions and vaccine hesitancy were significant, negative predictors of intentions, with a small effect size, also as expected. However, political orientation and free will beliefs did not significantly predict intentions. There were significant negative total indirect effects of vaccine hesitancy and political orientation, and a significant positive total indirect effect of free will beliefs, on intentions. Th e lack of residual direct effects of political orientation and free will beliefs on intention indicated full mediation of these effects by the social cognition beliefs, while the residual effect of vaccine hesitancy was indicative of partial mediation. However, the mediation proportion statistic (*P*_M_; Ditlevsen et al., 2005) indicated that a substantive proportion of the total effect of vaccine hesitancy on intention was directed through the social cognition constructs (*P*_M_ = .830). Specific indirect effects revealed that all of the social cognition beliefs contributed to the mediated effects of vaccine hesitancy, political orientation, and free will beliefs on intention, with the exception of the effect of free will beliefs through risk perceptions. Of the sociodemographic variables included as covariates in the model, only age had a statistically significant, negative effect on intentions with a small effect size. However, given that age was uncorrelated with intention, we surmised that this represents a suppressor effect. Overall, the model accounted for substantive variance in booster vaccine intentions (*R* ^2^ = .751).^2^ ^,^ ^3^

**TABLE 2:** Standardised parameter estimates for indirect effects for the structural equation model of the integrated model

Effect	*β*	95% CI		Effect	*β*	95% CI	
		LB	UB			LB	UB
Direct effects				Hesitancy → RP	.488^***^	.415	.562
Att → Int	.438^***^	.361	.514	Pol. orient. → RP	.297^***^	.209	.385
SN → Int	.275^***^	.183	.366	Free will → RP	−.126^**^	−.212	−.040
PBC → Int	.156^*^	.059	.253	Indirect effects			
RP → Int	−.100^**^	−.180	−.020	Hesitancy → Att → Int	−.141^***^	−.185	−.096
Hesitancy → Int	−.063^**^	−.126	.000	Pol. orient. → Att → Int	−.090^***^	−.134	−.045
Pol. orient. → Int	−.019	−.084	.046	Free will → Att → Int	.114^***^	.070	.159
Free will → Int	.012	−.051	.074	Hesitancy → SN → Int	−.078^***^	−.112	−.043
Age → Int	−.060^**^	−.115	−.005	Pol. orient. → SN → Int	−.057^*^	−.088	−.025
Gender → Int	−.036	−.087	.014	Free will → SN → Int	.096^***^	.056	.136
Education → Int	.023	−.027	.074	Hesitancy → PBC → Int	−.041^*^	−.070	−.011
Employ. → Int	.023	−.029	.074	Pol. orient. → PBC → Int	−.036^**^	−.064	−.008
Ethnicity → Int	.002	−.049	.054	Free will → PBC → Int	.062^*^	.020	.104
Status → Int	−.021	−.075	.032	Hesitancy → RP → Int	−.049^**^	−.089	−.009
Flu shot → Int	.033	−.019	.085	Pol. orient. → RP → Int	−.030^**^	−.055	−.004
Hesitancy → Att	−.321^***^	−.405	−.238	Free will → RP → Int	.013	−.001	.026
Pol. orient. → Att	−.205^***^	−.300	−.110	Sum of indirect effects^a^			
Free will → Att	.261^***^	.172	.351	Hesitancy → Int	−.308^***^	−.379	−.236
Hesitancy → SN	−.283^***^	−.366	−.200	Pol. orient. → Int	−.212^***^	−.287	−.136
Pol. orient. → SN	−.206^***^	−.300	−.112	Free will → Int	.285^***^	.213	.357
Free will → SN	.350^***^	.263	.436	Total effects^b^			
Hesitancy → PBC	−.260^***^	−.352	−.168	Hesitancy → Int	−.371^***^	−.449	−.292
Pol. orient. → PBC	−.231^***^	−.334	−.128	Pol. orient. → Int	−.231^***^	−.321	−.141
Free will → PBC	.399^***^	.305	.492	Free will → Int	.297^***^	.213	.381

^a^ Sum of indirect effects of through all model constructs.

^b^ Total effect comprising sums of all indirect effects through model constructs plus the direct effect; *β* = standardised parameter estimate; 95% CI = 95% confidence interval of standardised parameter estimate; LB = lower bound of 95% CI; UB = upper bound of 95% CI; Att = attitude; Int = intention; SN = subjective norm; PBC = perceived behavioral control; RP = risk perceptions; Hesitancy = COVID‐19 booster vaccine hesitancy; Pol. orient. = political orientation; Free will = free will beliefs; Education = dichotomous education level covariate; Employ. = dichotomous employment status covariate; Ethnicity = dichotomous race/ethnicity covariate; Status = previous positive test for COVID‐19 infection covariate; Flu shot = received an influenza vaccine in the past year covariate.

^*^ *p* < .05.

^**^ *p* < .01.

^***^ *p* < .001.

## DISCUSSION

The present study examined relations among social cognition constructs, individual difference and dispositional variables, and intentions with respect to receiving a COVID‐19 booster vaccine in a sample of previously vaccinated US residents. We tested an integrated model in which effects of key individual difference and dispositional variables, namely, vaccine hesitancy, political orientation, and free will beliefs, on individuals' booster vaccine intentions were mediated by social cognition constructs, namely, attitude, subjective norms, perceived behavioral control, and risk perceptions. Results of our model test indicated that, as predicted, all social cognition constructs exhibited statistically significant effects on booster vaccine intentions with small‐to‐medium effect sizes, and that these constructs completely mediated effects of political orientation and free will beliefs on intentions, and partially mediated effects of vaccine hesitancy.

Our findings provide the first evidence of the theory‐based constructs that are associated with vaccinated individuals' intentions to get a COVID‐19 booster vaccine. Attitudes and subjective norms had the largest effect sizes, indicating that individuals' beliefs in the value and utility of the booster vaccine, and the views of others' who are important to them, feature most prominently in informing estimates of their intentions. This corroborates previous research in the context of getting an initial COVID‐19 vaccine, suggesting that individuals are both utilitarian and account for the views of others they deem most significant, most likely their immediate family and friends (e.g. Caserotti et al., 2021; Guillon & Kergall, 2021), in their decision making. Perceptions of control and risk perceptions were also influential but less so by comparison. This contrasts with previous research on the initial vaccine in which vaccine risk perceptions and self‐efficacy featured more prominently (Karlsson et al., 2021; Tong et al., 2021). It may be the case that vaccinated individuals have previously formed, and acted on, intentions to perform an identical behavior, so perceptions of the inherent risks of getting vaccinated may have been allayed, and confidence in getting the vaccine enhanced, as a result of the previous experience and vaccine availability, reducing the extent to which they inform subsequent vaccination decisions.

A key innovation of the current study was the inclusion of vaccine hesitancy, political orientation, and free will beliefs in the proposed integrated model—key individual difference and dispositional belief‐based variables expected to be implicated in decisions to get a COVID‐19 booster vaccine. Consistent with our predictions, these variables were related to intentions, but their effects were largely or, in the case of political orientation and free will beliefs, entirely accounted for by the proximal social cognition constructs, attitudes, subjective norms, perceived behavioral control, and risk perceptions, from which people draw when estimating their vaccine intentions. These findings corroborate the theoretical perspective that these constructs serve as sources of information when individuals estimate their beliefs with respect to getting a booster vaccine when offered in future (see Ajzen, 1991; Kaushal et al., 2021). They also extend research that has shown these individual difference and dispositional constructs to be related to intentions to get an initial COVID‐19 vaccine (e.g. Bilewicz & Soral, 2021; Guillon & Kergall, 2021) by demonstrating a potential mechanism by which they inform intentions, that is, by informing the beliefs that underpin intentions. The direct effects of the individual difference and dispositional constructs on the social cognition constructs were nontrivial and substantive, indicating that they are influential in decision‐making.

But it is important to note that that these effects also imply that these constructs are not the only bases on which individuals estimate their vaccination intentions. Other unmeasured social cognition constructs might account for unique variance in intentions such as affective (e.g. anticipated regret or guilt), normative (e.g. moral norms), and non‐conscious (e.g. implicit attitudes or motives) beliefs, which have been shown to be related to health behaviors (e.g. Hagger et al., 2017; Keatley et al., 2012) including in the context of COVID‐19 (e.g. Chou & Budenz, 2020; Hagger, Smith, et al., 2020). Similarly, other unmeasured sociodemographic variables may also have been related to intentions, such knowledge of having a chronic disease or underlying medical condition. Individuals with certain illnesses or underlying medical conditions may have a higher risk of COVID‐19 infection and may have more severe consequences if infected (CDC, 2021c). Awareness of these elevated risks among individuals with these illnesses or conditions will likely affect their booster vaccine intentions.

Finally, the nontrivial, albeit small, residual effect of vaccine hesitancy on booster vaccine intentions that was unaccounted for by the social cognition constructs suggest that the current model was insufficient in accounting for all of the variance shared between hesitancy and intention. Again, other unmeasured constructs may mediate this association. For example, threat and coping appraisals from protection motivation theory, such as perceived severity and vulnerability, and perceived efficacy of the booster vaccine, respectively, may have accounted for effect of vaccine hesitancy on intentions (see Tong et al., 2021 for an example). Similarly, the affective beliefs individuals hold with respect to their booster vaccination intentions such as fears or concerns about vaccine procedures themselves (e.g. fear of injections and needles and negative prior experiences with vaccination) may also affect intentions (Freeman et al., 2021; Giuliani et al., 2021). Such beliefs may not have been adequately encompassed by the attitude or risk perception constructs in the current study because they do not relate directly to the specific behavior but may be reflected in vaccine hesitancy and feed forward to individuals' estimates of their booster vaccine intentions. We look to future research to examine a broader portfolio of social cognition and belief‐based mediators that more directly reflect vaccine procedures to verify this speculative explanation.

The innovation of the current findings is that they provide the first evidence of the social cognition constructs that relate to booster vaccine intentions. These findings are congruent with findings of prior research applying the theory of planned behavior to predict vaccination intentions for a number of illnesses (e.g. Gerend & Shepherd, 2012; Ng et al., 2020), including COVID‐19 (e.g. Caserotti et al., 2021; Guillon & Kergall, 2021). However, while such research may partly be informative of expected pattern of effects for these constructs across vaccination contexts, mere extrapolation of findings to intentions to get a COVID‐19 booster vaccine would fail to account for the specific contextual circumstances that determine booster vaccine decision‐making. In other words, even though the underlying theoretical determinants of vaccination‐related intentions may be similar, there are likely important context‐specific considerations that affect individuals' vaccine intentions in specific contexts like getting a booster vaccine. Such considerations may include concerns and beliefs that have been adjusted in response to information about, and experience with, the virus in the interim between initial vaccination and eligibility for a booster. These might include beliefs that the vaccine lacks efficacy based on observed breakthrough infections, reduced perceptions of risk and perceived severity of the illness over time, and elevated anxiety over the administration of the vaccine itself after prior experience (Finney Rutten et al., 2021). While we did not explicitly measure these beliefs, they are likely considerations that participants would have taken into account when prompted to estimate their attitudes, subjective norms, perceived behavioral control, and intentions toward the booster vaccine. The current study is the first to provide such data and stands as the initial contribution to building an evidence base of social cognition booster vaccine correlates, which we expect to be further augmented as subsequent studies on booster vaccine intentions are disseminated.

Alongside these findings, the current research is also uniquely informative of the processes by which more stable, generalized constructs inform the decision‐making process that, according to the theory of planned behavior, precedes decision‐making. Our findings suggest that social cognition constructs are capable of accounting for the effects of these stable, generalized beliefs on booster vaccine intentions. In the context of vaccine concerns driven by misinformation campaigns and conspiracy theories that are widely disseminated via populist press and social media, vaccine decision‐making is increasingly subject to political orientation, beliefs in free will, and vaccine hesitancy, all of which summarize generalized skepticism relating to vaccines and COVID‐19 mitigation more broadly (Finney Rutten et al., 2021). So, while it is not surprising that such generalized beliefs serve to inform more specific sets of beliefs that relate to intentions to get a booster vaccine, the mediation of generalized beliefs by the more specific beliefs is important given that the latter are likely more subject to change through information‐driven intervention efforts. Current findings, therefore, have value in signaling the belief‐based constructs that may be the most viable targets for campaigns to promote booster vaccine intentions.

### Implications for intervention

Current findings provide preliminary bases for interventions aimed at promoting booster vaccination intentions, particularly in the messages promulgated in information campaigns. Attitudes and subjective norms, as the most prominent determinants of booster vaccine intentions, and as mediators of the effects of vaccine hesitancy, political orientation, and free will beliefs on booster vaccine intentions, seem to be the most viable constructs to target in these messages. The value of these findings should not be trivialized given that promoting intentions by targeting change in more generalized beliefs through intervention are likely less effective because changing such entrenched beliefs is difficult. To illustrate, intervention attempts by health authorities and organisations that directly confront vaccine hesitancy with the goal of promoting vaccine uptake have been met with only modest success and small effect sizes (European Centre for Disease Prevention and Control [ECDC], 2017). This is also consistent with research on other generalized, more stable constructs such as personality. Although such dispositions are subject to change (e.g. Roberts et al., 2017; Siegler et al., 2021), it is not easy to do so, and effect sizes are often small. By contrast, targeting the specific beliefs that directly inform decisions to perform the target behavior, such as the beliefs that underpin intentions (e.g. attitudes, subjective norms, and perceived behavioral control) according to the theory of planned behavior, are likely to be more effective because such beliefs are less entrenched and are more sensitive to behavior change strategies such as persuasive communications (Hagger, Cameron, et al., 2020; Hamilton & Johnson, 2020). This is corroborated by research in the health domain that interventions using techniques to change attitudes, subjective norms, and perceived behavioral control or self‐efficacy lead to behavior change (Sheeran et al., 2016).

Our findings provide initial evidence that behavior‐specific beliefs may be viable targets for interventions aimed at promoting booster vaccine intentions than generalized, more stable beliefs. As a consequence, interventionists may consider using messages that highlight the utility and benefits of getting the COVID‐19 vaccine booster, consistent with intervention research adopting behavior change techniques targeting attitude change (Hamilton & Johnson, 2020), as well as emphasizing the importance of significant others as advocates of getting a booster vaccine, consistent with studies adopting techniques targeting normative belief change (Hagger, Cameron, et al., 2020; Sheeran et al., 2016). Such an approach may also be more effective than attempts targeting change in stable, generalized beliefs, such as vaccine hesitancy, because it may minimize psychological reactance to challenges to entrenched, strongly valued beliefs (Brinson, 2022). However, given the study design we are loathe to make specific recommendations for intervention, as current data do not imply causal effects or model change in intentions.

### Strengths, limitations, and avenues for future research

The current study is the first to provide evidence of the social cognition and individual difference correlates of COVID‐19 booster vaccine intentions. A further strength is the application of an innovative integrated model based on prior social cognition theories applied to health behavior to investigate associations between distal and proximal correlates of vaccine intentions. However, it is important to note some limitations against which the current findings should be interpreted.

First, our sample was not recruited using random selection or stratification based on sociostructural variables other than age and gender. We cannot, therefore, reliably generalize the current findings to the broader population. This is particularly the case given that our sample was more educated, had higher income, and were more likely to be White/Caucasian relative to the national population. Future studies should seek to replicate the predictions of the current model in samples that are more representative of the national population and also seek to verify these effects in underserved subgroups such as groups of individuals with lower education levels, on lower incomes, and from minority racial and ethnic backgrounds.

Second, we tested our model using a cross‐sectional, correlational design, which precludes any inference of causality in the estimated effects among the individual difference and dispositional variables, social cognition constructs, and booster vaccine intentions. It should, therefore, be stressed that the causal direction of the effects in our model are inferred from theory not the data. This means that other equally plausible models may exhibit good fit with the current data from a statistical perspective, even though they may not be theoretically coherent. In addition, it does not rule out the possibility that other unmeasured constructs may explain the estimated direct and mediated effects in our model. This also means that we do not make definitive recommendations for interventions based on the current data because we cannot affirm that interventions using techniques that target change in specific booster vaccine beliefs, such as attitudes and subjective norms, will lead to concomitant change in intentions. Panel designs that demonstrate cross‐lagged effects among model variables over time, or experimental or intervention studies that employ communication techniques that change beliefs and observe their effects on vaccine intentions, may provide data that better inform directional and causal effects. Nevertheless, it does not mean that the current data are not informative. At the bare minimum, they demonstrate the extent to which shared variance between the individual difference and dispositional variables and booster vaccine intentions are also shared with the social cognition constructs. They also provide a preliminary platform for variable selection in future research using the aforementioned designs and should also serve as a starting point for the development of an evidence base of determinants of booster vaccine intentions.

Finally, we did not include a direct measure of booster vaccine uptake. Although intentions have been shown to be a consistent correlate of health behavior (McEachan et al., 2011), the small‐to‐medium effect sizes for intention–behavior relations across multiple studies indicate that intentions should not be viewed as a proxy for behavior. So, while there is substantive value in identifying the correlates of booster vaccine intentions given that they are implicated in behavioral enactment, there is a need for the current preliminary findings to be extended to the prediction of actual uptake. Such research would verify the extent to which vaccine intentions predict subsequent behavior and perhaps signal the necessity for additional intervention strategies that might promote intention enactment outlined in dual phase models of behavior, such as promoting action and coping planning (Hagger & Luszczynska, 2014).

### Conclusion

COVID‐19 booster vaccine programs are likely to make an important contribution to managing the current pandemic and containing infection rates in a postpandemic world. However, as with many infection mitigation strategies, booster vaccine effectiveness is dependent on widespread uptake, and efficacious messaging campaigns aimed at promoting uptake should accompany vaccination programs in order to maximize compliance. Such campaigns should be informed by behavioral science research, particularly studies aimed at identifying potential targets for messaging interventions. The current research provided preliminary evidence to that end and should pioneer the building of a formative evidence base for intervention content as part of booster vaccine campaigns going forward. Our research has identified potential malleable belief‐based constructs that could be targeted in behavior change interventions to promote booster vaccine intentions, particularly attitudes and subjective norms, and, to a lesser extent, perceptions of control and risk. Our findings not only highlight potential theory‐based correlates that may form the target for intention messages but also indicate that targeting such beliefs may circumvent the potentially detrimental effects of more enduring beliefs such as vaccine hesitancy and political orientation on intentions. However, we stress the preliminary nature of these data and the need for future research that corroborate these findings more broadly and research that extends them to prediction of actual uptake and application to samples that are more representative of the broader population.

## CONFLICT OF INTEREST

The authors have no conflict of interest to declare.

## ETHICS STATEMENT

Approval for study procedures was granted prior to data collection from the Griffith University Research Ethics Committee (Reference #2021/108).

## Supporting information


**Data S1.** Supporting Information

## Data Availability

Data files, analysis scripts and output, and supplemental materials are available online: https://osf.io/emgn6/.

## ENDNOTES

^1^
Full parameter estimates including factor covariances and effects of covariates are provided in Appendix D in the Supporting Information.

^2^
Some participants reported receiving only one dose of the vaccine. The majority of these received the Johnson & Johnson/Janssen vaccine, a one‐shot regimen. However, a small number of participants indicated that they received one dose of the Pfizer (*n* = 2) and Moderna (*n* = 3) vaccines or selected “prefer not to say” (*n* = 2). We conducted a sensitivity analysis estimating our proposed model in data sets that included or excluded these participants (*n* = 7). As expected, the pattern of effects was unchanged—model fit statistics and effect size and statistical significance coefficients indicated trivial differences across the analyses. As a consequence, we have retained the original analysis. Analysis script and output for the sensitivity analysis is available online: https://osf.io/emgn6/.

^3^
Given that there was a small effect of vaccine regimen (“one shot” vs. “two shot”) on booster vaccine intentions, albeit not statistically significant, we thought it prudent to verify whether vaccine regimen had any effect on the variance explained in booster vaccine intentions in our model. We therefore conducted a supplementary analysis in which vaccine regimen was entered as a covariate in our structural equation model. The analysis revealed that the effect of the covariate was not statistically significant and had minimal effect on model fit statistics and on the effect size and statistical significance of the coefficients. We therefore retained our original analysis. Analysis script and output for the supplementary analysis is available online: https://osf.io/emgn6/.
