# Supplementary material for: Predicting COVID‐19 booster vaccine intentions
Source: Appl Psychol Health Well Being. 2022 Feb 22:10.1111/aphw.12349. Online ahead of print. doi: 10.1111/aphw.12349 (PMC9111247; doi:10.1111/aphw.12349)
Supplement: Supplementary file 1 — Data S1. Supporting Information [file APHW-9999-0-s001.docx]

Appendix A

*Items and Response Scales for Variables of the Integrated Model*

| Variable | Item(s)/measure | Scale |
| --- | --- | --- |
| Attitude | Getting a COVID-19 booster vaccine when it is offered to me would be… | 1 = bad, 7 = good  1 = worthless, 7 = valuable  1 = harmful, 7 = beneficial |
| Subjective norm | Most people who are important to me would approve of me getting a COVID-19 booster vaccine when it is offered to me.  Those people who are important to me think that I should get a COVID-19 booster vaccine when it is offered to me.  Of the people important to me, most would get a COVID-19 booster vaccine when it is offered to them. | 1 = strongly disagree, 7 = strongly agree |
| Perceived behavioral control | It is mostly up to me whether I get a COVID-19 booster vaccine when it is offered to me.  I am confident I can get a COVID-19 booster vaccine when it is offered to me.  I have complete control over whether I get a COVID-19 booster vaccine when it is offered to me. | 1 = strongly disagree, 7 = strongly agree |
| Risk perceptions | It would be risky for me to get a COVID-19 booster vaccine when it is offered to me.  If I got a COVID-19 booster vaccine when it is offered to me there would be risk involved. | 1 = strongly disagree, 7 = strongly agree |
| Vaccine hesitancy | Overall, how hesitant are you about getting a COVID-19 booster vaccine? | 1 = not at all, 7 = very much |
| Free will beliefs | Please respond to the following statements:  People always have the ability to do otherwise.  People always have free will.  How people’s lives unfold is completely up to them.  People ultimately have complete control over their decisions and their actions.  People have free will even when their choices are completely limited by external circumstances. | 1 = strongly disagree, 7 = strongly agree |
| Political orientation | How would you describe your political orientation?  How would you place your views on this scale, generally speaking? Political ideology. | 0% = strong liberal, 100% = strong conservative  0% = far left, 100% = far right |
| Intention | I intend to get the COVID-19 vaccine when it is offered to me.  I plan to get the COVID-19 vaccine when it is offered to me.  It is likely I will get the COVID-19 vaccine when it is offered to me. | 1 = strongly disagree, 7 = strongly agree |

Appendix B

*Descriptive Statistics and Reliability Estimates for the Integrated Model Variables*

| Construct | *M* | *SD* | Scale | Items | ω |
| --- | --- | --- | --- | --- | --- |
| Attitude | 5.995 | 1.209 | 1-7 | 3 | .924 |
| Subjective norm | 5.921 | 1.191 | 1-7 | 3 | .937 |
| Perceived behavioral control | 6.228 | 0.827 | 1-7 | 3 | .791 |
| Risk perceptions | 2.753 | 1.588 | 1-7 | 2 | .868 |
| COVID-19 booster vaccine hesitancy | 2.238 | 1.507 | 1-7 | 1 | − |
| Political orientation | 49.289 | 25.383 | 0-100 | 2 | .888 |
| Free will beliefs | 5.339 | 1.080 | 1-7 | 5 | .911 |
| Intention | 5.995 | 1.209 | 1-7 | 3 | .976 |

*Note*. ω = McDonald’s (1999) total omega reliability coefficient.

Appendix C

# *Correlations Among Integrated Model Variables*

|  | 1 | 2 | 3 | 4 | 5 | 6 | 7 | 8 | 9 | 10 | 11 | 12 | 13 | 14 |
| --- | --- | --- | --- | --- | --- | --- | --- | --- | --- | --- | --- | --- | --- | --- |
| 1. Intention | − |  |  |  |  |  |  |  |  |  |  |  |  |  |
| 2. Attitude | .755^***^ | − |  |  |  |  |  |  |  |  |  |  |  |  |
| 3. SN | .721^***^ | .625^***^ | − |  |  |  |  |  |  |  |  |  |  |  |
| 4. PBC | .600^***^ | .503^***^ | .614^***^ | − |  |  |  |  |  |  |  |  |  |  |
| 5. Risk perceptions | -.492^***^ | -.437^***^ | -.419^***^ | -.367^***^ | − |  |  |  |  |  |  |  |  |  |
| 6. Hesitancy | -.401^***^ | -.351^***^ | -.314^***^ | -.273^***^ | .521^***^ | − |  |  |  |  |  |  |  |  |
| 7. Pol. orient. | -.223^***^ | -.197^***^ | -.168^***^ | -.159^***^ | .339^***^ | .236^***^ | − |  |  |  |  |  |  |  |
| 8. Free will | .222^***^ | .180^***^ | .264^***^ | .279^***^ | -.031 | .027 | .242^***^ | − |  |  |  |  |  |  |
| 9. Age | -.003 | .012 | .037 | .041 | -.146^**^ | -.184^***^ | .123^**^ | -.056 | − |  |  |  |  |  |
| 10. Gender | -.030 | -.030 | -.001 | .009 | -.065 | -.024 | -.138^**^ | -.064 | -.140^**^ | − |  |  |  |  |
| 11. Education | .114^*^ | .060 | .097^*^ | .173^***^ | -.050 | -.046 | -.054 | .059 | -.001 | .016 | − |  |  |  |
| 12. Employment | .029 | .025 | -.007 | .030 | -.005 | .006 | .041 | -.035 | .210^***^ | -.080 | .084 | − |  |  |
| 13. Race/ethnicity | .073 | .064 | .078 | .128^**^ | -.048 | -.039 | .077 | -.021 | .196^***^ | -.019 | .107^*^ | .166^***^ | − |  |
| 14. COVID-19 status | .088 | .079 | .080 | .096 | -.299^***^ | -.236^***^ | -.205^***^ | -.134^**^ | .168^***^ | .007 | .058 | -.046 | .009 | − |
| 15. Flu shot | .185^***^ | .175^***^ | .175^***^ | .137^**^ | -.037 | -.102^*^ | .070 | .092^*^ | .140^**^ | -.029 | .032 | .105 | .115^*^ | -.062 |

*Note*. SN = Subjective norm; PBC = Perceived behavioral control; Hesitancy = COVID-19 booster vaccine hesitancy; Pol. Orient. = Political orientation; Free will = Free will beliefs; Education = Dichotomous education level covariate; Employment = Dichotomous employment status covariate; Race/ethnicity = Dichotomous race/ethnicity covariate; COVID-19 status = Previous positive test for COVID-19 infection covariate; Flu shot = Received an influenza vaccine in the past year covariate.

^***^ *p* < .001 ^**^ *p* < .01 ^*^ *p* < .05

Appendix D

*Full Standardized Parameter Estimates with Confidence Intervals for the Structural Equation Model of the Integrated Model*

| Effect | Estimate | 95% CI |  |  | Effect | Estimate | 95% CI |  |
| --- | --- | --- | --- | --- | --- | --- | --- | --- |
|  |  | LB | UB |  |  |  | LB | UB |
| Indicator loadings |  |  |  |  | Pol. orient.↔Education | -.060 | -.155 | .034 |
| Att | .961^***^ | .956 | .966 |  | Pol. orient.↔Employ. | .041 | -.053 | .136 |
| SN | .968^***^ | .964 | .972 |  | Pol. orient.↔Ethnicity | .076 | -.018 | .171 |
| PBC | .889^***^ | .874 | .904 |  | Pol. orient.↔Status | -.225^***^ | -.315 | -.134 |
| RP | .931^***^ | .922 | .940 |  | Pol. orient.↔Flu shot | .071 | -.024 | .165 |
| Pol. orient. | .944^***^ | .937 | .951 |  | Free will↔Age | -.056 | -.150 | .037 |
| Free will | .954^***^ | .948 | .960 |  | Free will↔Gender | -.067 | -.161 | .026 |
| Int | .988^***^ | .986 | .989 |  | Free will↔Education | .066 | -.027 | .160 |
| Direct effects |  |  |  |  | Free will↔Employ. | -.035 | -.128 | .059 |
| Att→Int | .438^***^ | .361 | .514 |  | Free will↔Ethnicity | -.015 | -.109 | .079 |
| SN→Int | .275^***^ | .183 | .366 |  | Free will↔Status | -.138^**^ | -.231 | -.046 |
| PBC→Int | .156^**^ | .059 | .253 |  | Free will↔Flu shot | .103^*^ | .010 | .196 |
| RP→Int | -.100^*^ | -.180 | -.020 |  | Age↔Gender | -.140^**^ | -.228 | -.052 |
| Hesitancy→Int | -.063^*^ | -.126 | .000 |  | Age↔Education | -.001 | -.090 | .089 |
| Pol. orient.→Int | -.019 | -.084 | .046 |  | Age↔Employ. | .210^***^ | .125 | .296 |
| Free will→Int | .012 | -.051 | .074 |  | Age↔Ethnicity | .196^***^ | .110 | .282 |
| Age→Int | -.060^*^ | -.115 | -.005 |  | Age↔Status | .168^***^ | .081 | .255 |
| Gender→Int | -.036 | -.087 | .014 |  | Age↔Flu shot | .140^**^ | .053 | .228 |
| Education→Int | .023 | -.027 | .074 |  | Gender↔Education | .016 | -.074 | .105 |
| Employ.→Int | .023 | -.029 | .074 |  | Gender↔Employ. | -.080 | -.169 | .009 |
| Ethnicity→Int | .002 | -.049 | .054 |  | Gender↔Ethnicity | -.019 | -.108 | .071 |
| Status→Int | -.021 | -.075 | .032 |  | Gender↔Status | .007 | -.082 | .097 |
| Flu shot→Int | .033 | -.019 | .085 |  | Gender↔Flu shot | -.029 | -.118 | .061 |
| Hesitancy→Att | -.321^***^ | -.405 | -.238 |  | Education↔Employ. | .084 | -.005 | .173 |
| Pol. orient.→Att | -.205^***^ | -.300 | -.110 |  | Education↔Ethnicity | .107^*^ | .018 | .195 |
| Free will→Att | .261^***^ | .172 | .351 |  | Education↔Status | .058 | -.031 | .147 |
| Hesitancy→SN | -.283^***^ | -.366 | -.200 |  | Education↔Flu shot | .032 | -.058 | .121 |
| Pol. orient.→SN | -.206^***^ | -.300 | -.112 |  | Employ.↔Ethnicity | .166^***^ | .079 | .253 |
| Free will→SN | .350^***^ | .263 | .436 |  | Employ.↔Status | -.046 | -.135 | .044 |
| Hesitancy→PBC | -.260^***^ | -.352 | -.168 |  | Employ.↔Flu shot | .105^*^ | .017 | .194 |
| Pol. orient.→PBC | -.231^***^ | -.334 | -.128 |  | Ethnicity↔Status | .009 | -.080 | .099 |
| Free will→PBC | .399^***^ | .305 | .492 |  | Ethnicity↔Flu shot | .115^*^ | .027 | .204 |
| Hesitancy→RP | .488^***^ | .415 | .562 |  | Status↔Flu shot | -.062 | -.151 | .028 |
| Pol. orient.→RP | .297^***^ | .209 | .385 |  | Indirect effects |  |  |  |
| Free will→RP | -.126^**^ | -.212 | -.040 |  | Hesitancy→Att→Int | -.141^***^ | -.185 | -.096 |
| Covariances |  |  |  |  | Pol. orient.→Att→Int | -.090^***^ | -.134 | -.045 |
| Att↔SN | .584^***^ | .516 | .652 |  | Free will→Att→Int | .114^***^ | .070 | .159 |
| Att↔PBC | .476^***^ | .386 | .567 |  | Hesitancy→SN→Int | -.078^***^ | -.112 | -.043 |
| Att↔RP | -.320^***^ | -.417 | -.223 |  | Pol. orient.→SN→Int | -.057^**^ | -.088 | -.025 |
| SN↔PBC | .624^***^ | .548 | .700 |  | Free will→SN→Int | .096^***^ | .056 | .136 |
| SN↔RP | -.316^***^ | -.413 | -.219 |  | Hesitancy→PBC→Int | -.041^**^ | -.070 | -.011 |
| PBC↔RP | -.292^***^ | -.403 | -.181 |  | Pol. orient.→PBC→Int | -.036^*^ | -.064 | -.008 |
| Hesitancy↔Pol. orient. | .250^***^ | .161 | .339 |  | Free will→PBC→Int | .062^**^ | .020 | .104 |
| Hesitancy↔Free will | .029 | -.065 | .122 |  | Hesitancy→RP→Int | -.049^*^ | -.089 | -.009 |
| Pol. orient.↔Free will | .268^***^ | .175 | .361 |  | Pol. orient.→RP→Int | -.030^*^ | -.055 | -.004 |
| Hesitancy↔Age | -.184^***^ | -.270 | -.097 |  | Free will→RP→Int | .013 | -.001 | .026 |
| Hesitancy↔Gender | -.024 | -.114 | .065 |  | Sum of indirect effects^a^ |  |  |  |
| Hesitancy↔Education | -.046 | -.136 | .043 |  | Hesitancy→Int | -.308^***^ | -.379 | -.236 |
| Hesitancy↔Employ. | .006 | -.084 | .095 |  | Pol. orient.→Int | -.212^***^ | -.287 | -.136 |
| Hesitancy↔Ethnicity | -.039 | -.129 | .050 |  | Free will→Int | .285^***^ | .213 | .357 |
| Hesitancy↔Status | -.236^***^ | -.320 | -.151 |  | Total effects^b^ |  |  |  |
| Hesitancy↔Flu shot | -.102^*^ | -.190 | -.013 |  | Hesitancy→Int | -.371^***^ | -.449 | -.292 |
| Pol. orient.↔Age | .125^*^ | .032 | .219 |  | Pol. orient.→Int | -.231^***^ | -.321 | -.141 |
| Pol. orient.↔Gender | -.147^**^ | -.240 | -.054 |  | Free will→Int | .297^***^ | .213 | .381 |

*Note*. ^a^Sum of indirect effects of through all model constructs; ^b^Total effect comprising sums of all indirect effects through model constructs plus the direct effect; Estimate = Standardized parameter estimate; 95% CI = 95% confidence interval of standardized parameter estimate; LB = Lower bound of 95% CI; UB = Upper bound of 95% CI; Att = Attitude; Int = Intention; SN = Subjective norm; PBC = Perceived behavioral control; RP = Risk perceptions; Hesitancy = COVID-19 booster vaccine hesitancy; Pol. orient. = Political orientation; Free will = Free will beliefs; Education = Dichotomous education level covariate; Employ. = Dichotomous employment status covariate; Ethnicity = Dichotomous race/ethnicity covariate; Status = Previous positive test for COVID-19 infection covariate; Flu shot = Received an influenza vaccine in the past year covariate.

^***^ *p* < .001 ^**^ *p* < .01 ^*^ *p* < .05
